# Probing the Metabolic Landscape of Plant Vascular Bundles by Infrared Fingerprint Analysis, Imaging and Mass Spectrometry

**DOI:** 10.3390/biom11111717

**Published:** 2021-11-18

**Authors:** André Guendel, Alexander Hilo, Hardy Rolletschek, Ljudmilla Borisjuk

**Affiliations:** Department of Molecular Genetics, Leibniz Institute for Plant Genetics and Crop Plant Research, 06466 Gatersleben, Germany; guendel@ipk-gatersleben.de (A.G.); hilo@ipk-gatersleben.de (A.H.); rollet@ipk-gatersleben.de (H.R.)

**Keywords:** FTIR spectroscopy, imaging, LC-MS, plant tissue extracts, chemometric fingerprinting, *Brassica napus*, phloem, xylem, vascular bundle

## Abstract

Fingerprint analysis is a common technique in forensic and criminal investigations. Similar techniques exist in the field of infrared spectroscopy to identify biomolecules according to their characteristic spectral fingerprint features. These unique markers are located in a wavenumber range from 1800 to 600 cm^−1^ in the mid infrared region. Here, a novel bioanalytical concept of correlating these spectral features with corresponding mass spectrometry datasets to unravel metabolic clusters within complex plant tissues was applied. As proof of concept, vascular bundles of oilseed rape (*Brassica napus*) were investigated, one of the most important and widely cultivated temperate zone oilseed crops. The link between mass spectrometry data and spectral data identified features that co-aligned within both datasets. Regions of origin were then detected by searching for these features in hyperspectral images of plant tissues. This approach, based on co-alignment and co-localization, finally enabled the detection of eight distinct metabolic clusters, reflecting functional and structural arrangements within the vascular bundle. The proposed analytical concept may assist future synergistic research approaches and may lead to biotechnological innovations with regard to crop yield and sustainability.

## 1. Introduction

The biometric analysis of fingerprints as a means with which to identify individuals is based on two basic ground truths: persistence and individuality. In other words, specific fingerprint characteristics do not change over time and are unique enough to identify a specific individual. The same principle can be applied to mid-infrared spectroscopy. The wavenumber range between 1800 and 600 cm^−1^ is commonly referred to as a spectral fingerprint region. Within this spectral range, a multitude of functional groups in biomolecules absorb infrared radiation, creating a unique combination of band shapes that allow for quick and automated compound/-group identification given that a reference library is established. Similar to fingerprint databases for forensic purposes, characteristic features are compared between a sample and a database, allowing for a correlative likelihood to be estimated insofar as the sample characteristics and database entry are identical. Currently, Fourier transform infrared (FTIR) spectroscopy has evolved into a valuable analytic tool with applications in various fields of science, including medicine [1,2], agriculture, food, and plant sciences [3,4]. Harnessing FTIR spectroscopy for the study of metabolite distribution inside plant tissues has opened new perspectives for high-resolution investigations into the vascular system of plants [5,6,7].

Both animals and higher plants possess vascular systems, which are the essential routes for long-distance transport within the organism (plant body) [8]. In humans, the vasculature is made up of blood vessels (arteries and veins, carrying blood throughout the body) and the thin-walled lymph vessels that carry lymphatic fluid. In plants, there are xylem vessels, which mainly transfer water and mineral compounds from roots to leaves, and there is phloem, which mainly transfers assimilates (such as sucrose) from photosynthetic organs (leaf) to roots, seeds, fruits, etc. In addition, numerous other components are transferred across the plant, such as mRNA, hormones, or proteins/peptides [8]. Although both types of vessels are collected in an individual vascular bundle, transport within the phloem and xylem vessels can occur in a reciprocal direction [9]. Large networks of such bundles connect distinct organs throughout the entire plant body [10]. The structure of plant vascular bundles can be investigated using microscopic observations, but biochemical composition and functionality are less accessible. Many efforts have been devoted to collecting phloem samples; current methods involve cutting/puncturing, aphid stylectomy, and EDTA-facilitated exudation [11,12,13,14,15]. The collected sap is normally analyzed by chromatography coupled to mass spectrometry (MS), allowing one to identify hundreds of individual compounds (metabolites, proteins, and others). However, these samples can be considered relatively inaccurate regarding their association with their origins. This is due to sampling techniques either lacking localized selectiveness or not being able to deliver sufficient extract amounts to allow for the proper detection of all desired compounds.

In current plant research, a number of questions on vascular functionality remain to be answered. One of the most important questions is related to the metabolic architecture of an individual vascular bundle [16,17]. A comprehensive answer requires one to define functional clusters based on the combined mapping of biochemical, molecular, and structural features. Although technologies such as matrix assisted laser/desorption ionization mass spectrometry imaging (MALDI-MSI) allow for localization in 2D (3D via image rendering) along with high selectivity in compound identification, these are not suitable for all types of biological samples [18]. Due to the composition of the biochemical make up of some plant tissues (high contrasts between hard and soft tissue regions), some unwanted matrix effects can occur, diminishing the capacity of the method for accurate localization and detection.

**Figure 1:**
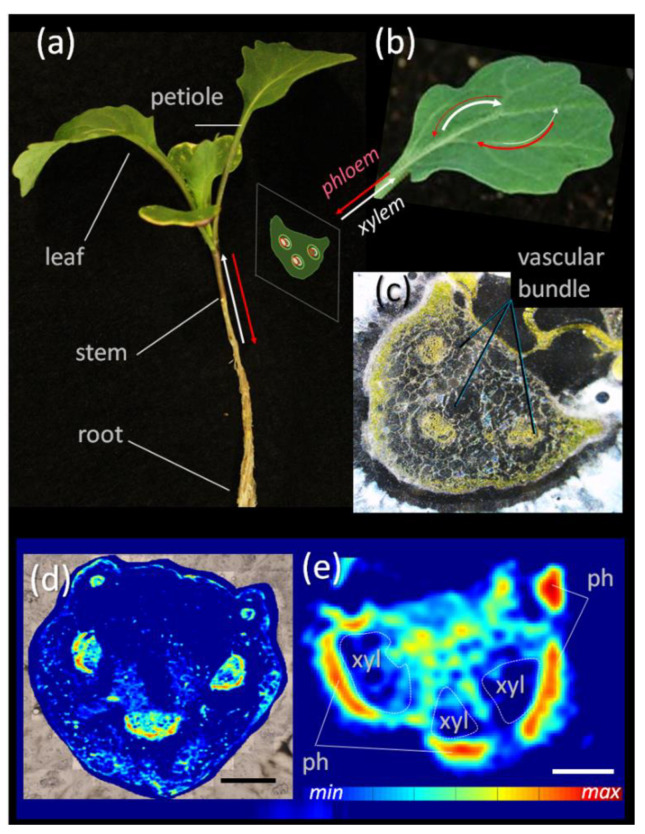
**Overview of *Brassica napus* petioles and their vascular features.** (**a**) Entire plant showing distinct organs at early development; (**b**) individual leaf with schematic representation of petiole cross-section; (**c**) microscopic image of a petiole cross-section showing the position of the three main vascular bundles; (**d**) sucrose map showing sucrose concentration (color-coded) across the petiole cross-section analysed by FTIR microspectroscopy; (**e**) enlarged view on an individual vascular bundle showing the preferential accumulation of sucrose in the phloem. Abbreviations: ph—phloem, xyl—xylem. Bar: 100 µm in (**d**) and 500 µm in (**e**).

Therefore, this study proposes a procedure that combines the high analytical power of chromatographic/MS methods with the localization capabilities of infrared imaging by fingerprint analysis. As a proof of concept, this study focuses on the analysis of phloem sap of *B. napus* leaf petioles (Figure 1a–c). Sap collection is often performed by simple cutting experiments, which do not allow for any kind of interpretation of the results towards the region of origin on a micro scale (e.g., tissue region). As cutting experiments often result in a relatively large access area, the entire region will be represented within the sample composition and not just smaller target areas within that cut. Here, the major spectral components of the extracted sap samples are assessed by principal component analysis and used to establish a spectral library of markers. Correlating the marker signal in a set of heterogenic sap samples with the respective high detail chemical composition (MS dataset) by hierarchical cluster analysis created associations between subsets of chemical components and spectral fingerprint features. When employing infrared imaging on tissue sections of similar samples, these fingerprint features were compared to the spectral features within individual pixels, creating a correlation map between a chemical compound group of the sap samples and the tissue regions. This novel approach established a link to the region of origin of the compound group and eventually uncovered metabolic clusters across the vascular bundle.

## 2. Materials and Methods

### 2.1. Plant Cultivation

*Brassica napus* plants (cultivar Athena) were grown under standard light conditions until the three-leaf stage. Samples were taken at 9 a.m. from two groups, with group #1 being the control and group #2 having been subjected to shading from 4 p.m. the day prior to sampling. Shading was applied to force chemical variability among sap samples. This forced the later analysis by PCA to promote the detection of sap-related chemical responses in main components and reduce noise influence from unrelated origins. Sap was taken as exudate according to the well-established puncturing procedure [15] at the petiole of leaf #2 from eight plants per group. Additionally, a standard set of five petiole tissue samples from group #1 was taken for imaging. Samples were snap frozen and stored at −80 °C until further processing.

### 2.2. Sample Preparation and LC-MS Analysis

For the untargeted metabolite analysis, the collected phloem sap samples were diluted with 50% (*v*/*v*) methanol followed by ion chromatography separation using an ICS-5000+HPIC system (Thermo Scientific, Dreieich, Germany) coupled to a Q-Exactive Plus hybrid quadrupol-orbitrap mass spectrometer (Thermo Scientific, Dreieich, Germany). For details, see [19].

### 2.3. FTIR Spectroscopy

Sap samples were dried, re-dissolved in MeOH (10 µL), and analyzed by ATR spectroscopy using a Bruker Invenio S (Bruker Optics, Ettlingen, Germany). The spectrometer was continuously purged with dry air. Spectra were recorded of 16 individual sap samples in duplicate in the IR range 4000–600 cm^−1^ with a resolution of 2 wavenumbers (WN). Prior to each measurement, a background spectrum of the clean sampling stage was taken. The dissolved sample was transferred onto the sampling stage with a 10 µL micro syringe and recrystallized when the solvent evaporated. Background features were subtracted from the sample spectra automatically during measurements using OPUS 8.5 (Bruker Optics). Spectral data were saved as MATLAB files (.mat, The MathWorks Inc., Novi, MI, USA) for further processing.

### 2.4. FTIR Imaging and Sample Preparation

Leaf petiole samples were frozen in liquid nitrogen and embedded in Tissue-Tek cryomolds using Tissue-Tek O.C.T. (Sakura Finetek) at −20 °C. Embedded tissues were cross sectioned (12 µm) with a cryotome CryoStar NX7 (Thermo Fisher Scientific, Dreieich, Germany) and transferred onto MMI membrane RNAse free slides (Molecular Machines & Industries, Eching, Germany). Tissue sections were lyophilized and stored in darkness at room temperature until analysis. The spectral membrane fingerprint of the slides was also used for internal standardization as described in [6].

Imaging was performed using a Hyperion 3000 FTIR microscope (Bruker Optics) coupled to an Invenio S FTIR spectrometer (Bruker Optics) with an internal mid-infrared source. The focal plane array detector (64 × 64 pixel) was used in transmission mode. The imaging system was purged with dry air continuously. FTIR images were recorded in the spectral range of 3900 cm^−1^ to 800 cm^−1^ at a spatial resolution of 11 µm and a spectral resolution of 6 cm^−1^ using 15× (5.5 µm digital resolution with 2 × 2 pixels binning) infrared magnification objectives (Bruker Optics). Each spectrum comprised 64 coadded scans. A reference of a single focal plane array window of the empty light path was acquired before image acquisition and automatically subtracted from the recorded image by OPUS software (Bruker Optics). Atmospheric absorptions of water vapor and CO_2_ were corrected by OPUS during image acquisition. Image files up to 2 GB were stored as OPUS files, whereas larger files had to be saved as ENVI files for data import into MATLAB.

### 2.5. Data Processing

MS: The acquired data were processed using the untargeted metabolomics workflow of Compound Discoverer 3.1 software (Thermo Scientific, Dreieich, Germany), as described in Radchuk et al. [19]. To correct the batch effect of acquired data, the extracted raw peak area values for each compound were normalized based on the repeated measurements of the pooled quality control sample, using the cubic spline algorithm. The resulting normalized peak area values (arbitrary units) were used to describe the relative abundance of each compound in separate sap samples. Normalized peak area scores for detected components were imported into MATLAB (2019b, The MathWorks Inc., Novi, MI, USA). Each component’s values were vector normalized using the zscore function in MATLAB to be able to process the data along with IR data.

**Figure 2:**
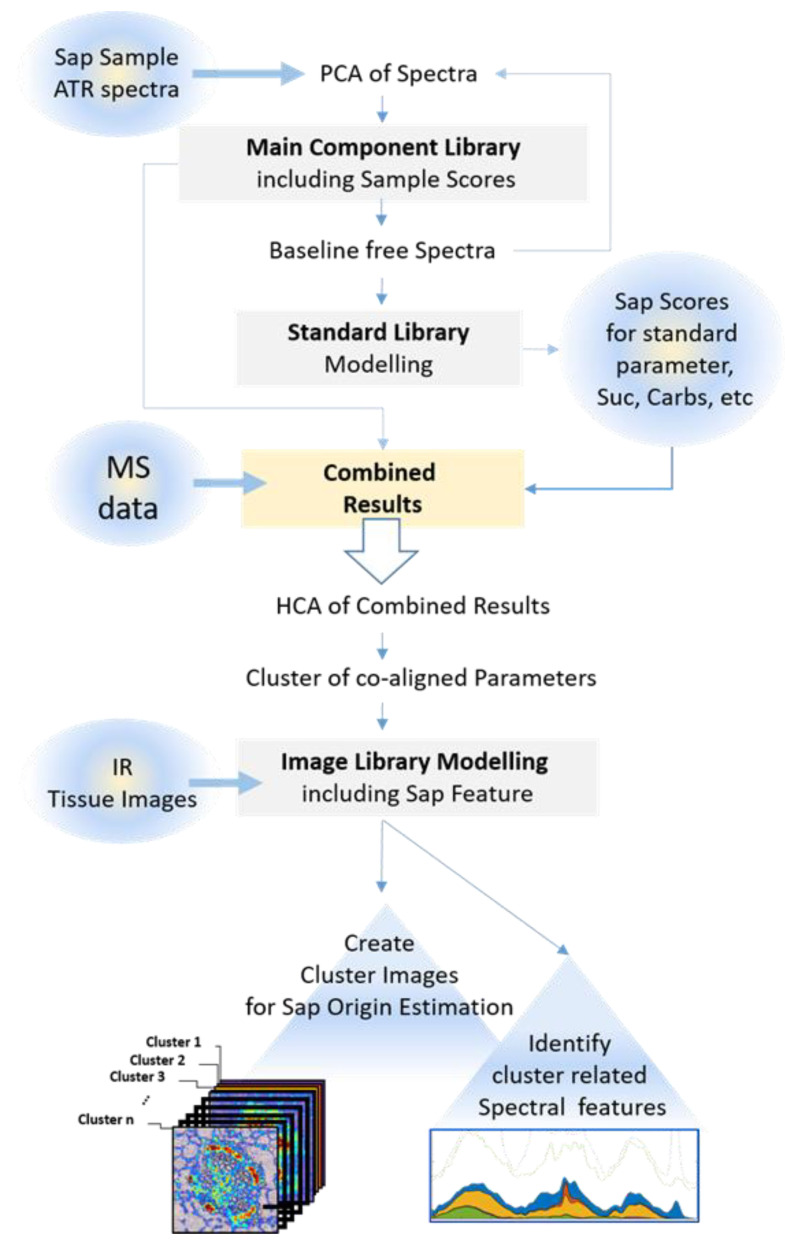
**Processing schematic of the fingerprint analysis.** This shows the integration and processing of FTIR spectral and imaging data together with related MS data to gain localization and characterization evidence (for detailed explanation, see text). A spectral component library is built from principal components of sap spectra. The scores of these library features are combined with MS peak area scores, normalized, and grouped into co-aligned parameters according to hierarchical clustering. This creates a link between MS components and modelled sap library components in tissue images.

FTIR data of sap spectra: Replicates of ATR sap spectra were loaded and combined in MATLAB and vector normalized. Baseline correction for image data was performed as depicted in Figure 2 and described for image spectra below. For sap spectra, baseline correction was minimal and was performed along with the principal component analysis (PCA) in two iterations on the sap data to create spectra of the main components, explaining 95% of the variance within the sample set fingerprint features. Measurement noise within relevant eigenvectors was reduced by incrementally remodeling noisy band shapes by a combination of linear fitted baseline and Gauss-fitted peak shapes (Supplementary Figure S1c). This procedure was essential to reduce the impact of environmental bands from water vapor introduced during spectrum acquisition. Therefore, IR bands such as Amide I and II were individually remodeled using up to 6 Gauss functions and a linear offset function in the local range of the noisy band shape (Equation (1)). These ‘noiseless’ main components were used to baseline correct the measured spectra, reduce noise (Supplementary Figure S1e), and perform the second iteration of PCA to create the final main component database. The second iteration was performed to compensate for any alterations in component scores and eigenvectors as a result of the denoising of the first iteration of PCA. This sap component library (Supplementary Figure S1g) was then integrated into the model in order to extract sap-related features from infrared tissue images.

(1) $$Abs_{spec}\left(WN\right)=\overset{n}{\underset{i=1}{\sum }}a_{i}\cdot \exp\left(-{\left(\frac{WN-b_{i}}{c_{i}}\right)}^{2}\right)+g\cdot WN+h$$

FTIR image data of petiole cross sections: For imaging data, OPUS files were imported into MATLAB by the irootlab toolbox [20] to further process hyperspectral images. Spectral features such as carbohydrates and sucrose fingerprints, along with baseline features, were extracted using an extended multiplicative signal correction (EMSC) model [21] adopted into an in-house-developed analytical MATLAB routine for statistical and quantitative spectral feature analysis, as described earlier [6]. The modeling routine was based on restricted partial linear least-squares regression to restrict spectral features to be non-negative. This procedure allows for the extraction of baseline variance-free fingerprint features for single and image spectra. Strictly viewed, the applied modeling approach is a combination of EMSC and multivariate curve resolution (MCR). In essence, both techniques can be used to extract spectral features from measured mixed spectra by remodeling single or multiple spectra in the form of Equation (2).

(2) M=CS+E

The variable M(Abs) represents the measured spectra matrix as a p × m matrix (p number of pixels/spectra × m number of wavenumbers). The variable C is the coefficient matrix (p × l number of unique component spectra), S the spectral component library (l × m), and the residual matrix is E (p × m). The difference between the two techniques is the composition of the CS complex. In an EMSC approach, the IR spectra M are fitted with a spectral library describing scatter features (e.g., Mie-scattering) by a library of functions describing baseline shapes combined with the average spectrum of M. The function S can be defined as a scatter function matrix of i baseline spectra and the average of M, thus creating a i + 1 × m dimensional matrix. By multiple iterations, it is possible to approximate the individual baseline variation of each sample spectra. The MCR technique would substitute the average of M in the matrix S with a library of j pure component spectra in the form of (I + j) × m in dimension. The baseline free matrix M_b_ is in either approach created according to Equation (3).

(3) $$M_{b}=M-\overset{i}{\underset{1}{\sum }}C_{i}S_{i}$$

(4) $$M_{b}=\overset{i+j}{\underset{i+1}{\sum }}C_{i}S_{i}+E$$

The applied procedure uses aspects of both EMSC and MCR. A preliminary subset of ~10% of the image spectra is computed by constrained linear least-squares regression (LSQ) according to MCR. It is noteworthy that, although model constraints increase quality, they also increase computation time. The resulting baseline free spectra M_b_ were then employed to baseline correct their representative subset in an EMSC model by substituting the I + 1 position in S with the best fitting M_b_ spectrum of the subset. Because the EMSC matrix S has fewer variables to fit than S in an MCR background, the computation is simplified without losing much accuracy. In the presented case, the model library consists of the sven major principal components for Mie scattering, as previously published [6], nine experimental components to compensate residual signal related to measurement corrections by OPUS (Bruker, Ettlingen) and a spectral library of 120 pure component spectra (Supplementary Table S1). In this study, a component is defined as a single chemical or a group of chemical structures that can be identified by a unique spectral signature/band composition or fingerprint. The 120 component spectra relate to 105 individual chemical structures. Spectral variability in single compounds due to treatment conditions is accounted for by including spectra of a pure compound both in its dry and dried state after lyophilization. This is especially important for carbohydrates, which undergo chemical shifts due to them drawing moisture to equilibrate with the surrounding environment. In essence, the MCR subset model is computed with an extensive number of variables (i = 16 baseline and j = 120 chemical variables), in contrast to the EMSC model based on the results of the subset, which reduces the model to only ~12% (i = 16 baseline and j = 1 chemical variable) of its original complexity and reduces computation time. Finally, the baseline-corrected spectra M_b_ were correlated with average pure component absorbances, as established by the CS term in Equations (4) and (5) by partial least-squares (PLS) regression. The resulting calibration model was applied to gain the pure deconvoluted component absorbances for the EMSC-computed M_b_ spectra. As the subset of representative 10% spectra analyzed by the complex MCR model was additionally modelled by the PLS calibration, both approaches were compared with each other by Pearson correlation to assess their performance factors, such as R, RMSE, and RPD (signal-to-noise estimator), within an internal validation (see Equations (6)–(8)).

(5) $$\bar{Abs_{cmpd\left(j\right)}}=\bar{\underset{j\in cmpd}{\sum }c_{j}S_{j}}$$

(6) $$R_{cmpd}=corr\left(\bar{Abs_{cmpd\left(j,PLS\right)}},\bar{Abs_{cmpd\left(j,MCR\right)}}\right);pixel\in MCR\cup^{\text{​}}PLS$$

(7) $$RMSE_{cmpd}=\sqrt{mean\left({\left(\bar{Abs_{cmpd\left(j,PLS\right)}}-\bar{Abs_{cmpd\left(j,MCR\right)}}\right)}^{2}\right)};\;pixel\in MCR\cup^{\text{​}}PLS$$

(8) $$RPD_{cmpd}=\frac{median\left(\bar{Abs_{cmpd\left(j,MCR\right)}}\right)}{RMSE_{cmpd}}$$

In order to establish similar absorbance coefficients for the sap component library spectra, the baseline free sap spectra and petiole image spectra were remodeled, again incorporating the 15 sap components into the library. All sap spectra and 10% subsets for images were remodeled by constrained LSQ regression that was used to create the PLS calibration as noted above in order to achieve consistent absorbance scores. Further relative importance of IR components can be established by determining the overall percentage of a compound’s absorption in relation to the total baseline free absorbance Abs_b_, as in Equation (9).

(9) $$\bar{rel.\;Abs_{cmpd\left(j\right)}}=\frac{\bar{Abs_{cmpd\left(j\right)}}}{\bar{Abs_{b}}}$$

MS and IR absorbance scores were compiled in a single variable matrix of the dimensions 16 sap samples × 92 variables (MS and IR). Each variable was seen as an individual component and was normalized by the zscore function; as such, they were mean centered and normalized by standard deviation. Hierarchical clustering was performed on this dataset using Euclidean distance computation and ‘Ward’-linking. Clustering was performed by set distance values for 10.6 and 16.5. These clustering thresholds were established by minima analysis of the cumulative distribution function (cdf) established for the ‘Ward’ joining distances. The two selected minima represent the first overall minima and the first absolute minima of the cdf.

## 3. Results and Discussion

### 3.1. Sucrose Mapping of Vascular Bundles

Sucrose is the main sugar in the phloem sap of *B. napus* [22]. Previous methodological developments in FTIR micro-spectroscopy allowed for the imaging of sucrose distribution across diverse plant tissues [6]. Here, this methodology was applied to visualize the sucrose distribution across petioles of *B. napus* plants (Figure 1). Sucrose was highly enriched within the vascular bundles of the petiole (Figure 1d). A closer inspection of the bundle (Figure 1e) revealed that the majority of sucrose was found within the phloem region of vascular bundles, but much less within the xylem and other parenchyma regions. Such a distribution pattern is in accordance with findings on other species and reflect the main function of phloem in sugar transport across the plant. Although this particular FTIR application can reliably visualize sucrose gradients, the distribution of other (low abundant) metabolites remains invisible. To overcome this limitation, we propose a combined use of data derived from mass spectrometry and FTIR micro-spectroscopy. The advanced, integrative approach is outlined below.

### 3.2. Experimental Design and Workflow of the Novel Procedure

**Figure 3:**
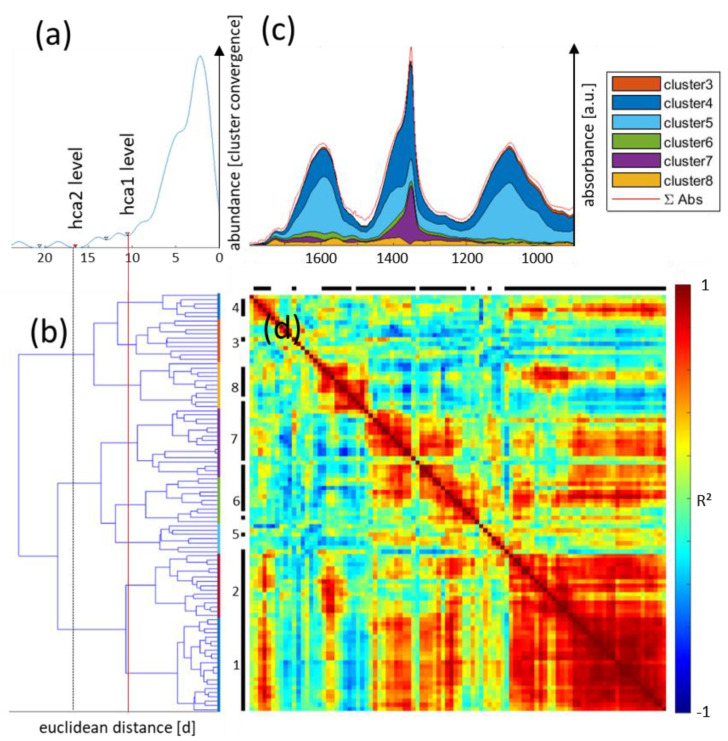
**Sap analysis according to the general workflow after principal components for spectral data were established.** (**a**) The distance level for hierarchical clustering (hca) was set according to the first minima of the cluster convergence distance histogram; (**b**) shows the corresponding dendrogram of MS and infrared scores for sap derived parameters, with colored bars representing cluster associations at the set distance level (hca1, red line); (**c**) representative sum spectra of all sap spectra (red line); the fingerprint region is deconvoluted into its cluster-related fingerprint spectra according to the legends color code; (**d**) correlation matrix of all MS and FTIR components included in the hca (color coded according to R² from negative (blue) to positive (red) correlation). Black pixel along the x- and y-axis show MS parameters, whereas white gaps are FTIR variables.

The basis of the procedure is depicted in Figure 2. First of all, the sap samples collected from *B. napus* plants were analyzed using both ATR spectroscopy and mass spectrometry. Following spectral analysis, the main chemical components detected by PCA/modeling of sap spectra (Supplementary Figure S2) and MS were compiled to create a final component database. Next, hierarchical cluster analysis identified the associations between (subsets of) chemical components (MS dataset) and spectral fingerprint features. When employing infrared imaging on tissue sections of similar samples, these fingerprint features were compared to the spectral features within individual pixels. This created a scores map of the IR fingerprint of the sap samples’ chemical compound clusters and tissue regions, thereby defining metabolic clusters across the vascular bundle. The important step of combining FTIR and MS data on sap composition is explained in Figure 3. The PCA scores of the 15 main sap components and the scores for the 5 standard sap features (carbohydrates, soluble sugars, hexoses, protein/aminoacids and other compounds; see Figure 2 and Supplementary Tables S1 and S2) were combined with the 77 metabolites (detected by MS; see Supplementary Tables S2–S4) for each sap sample, creating a mixed dataset of infrared main component scores and peak areas. The representative data were then normalized by vector normalization for each component. The normalized 92 components were then subjected to hierarchical cluster analysis using an Euclidean distance matrix and the ‘Ward’ linkage method. Analysis of the cumulative distribution function (cdf) of the linking distance histogram identified a distance of 10.6 as the first minima in this cdf (Figure 3a,b). The data were clustered according to this critical distance into eight clusters (hca1). Additionally, a critical clustering distance of 16.5 resulted in an optimal classification, with three clusters (hca2) combining co-aligned features from MS and IR data. Further analysis was performed solely using the hca1 grouping (red line; Figure 3b), because hca2 (black dashed line; Figure 3b) resulted in too general a feature combination and would not enable a detailed image analysis for the detection of differential cluster image regions. This is evident when evaluating the displayed correlation matrix for Pearson correlation of the individual MS and IR components (Figure 3d). If clustered at the hca2 level, the clusters containing clusters 5, 6, and 7, or 3, 4 and 8, would be less homogenous than at distance level of hca1.

The clusters according to hca1, including infrared features, were used as a reference set to compare cluster related fingerprint features from the hyperspectral images of petiole tissue in an effort to derive likely origin patterns for co-aligned MS sap features within the same cluster, as depicted in the final steps of Figure 2. These features were reconstructed using the previously determined PCA loading spectra as additional spectra according to the performed EMSC procedure. For IR images and spectra, the average absorbance in the range of 1800–900 cm^−1^ is represented as the components’ score values. The resulting average sap fingerprint is shown for selected clusters as colored areas in Figure 3c.

### 3.3. Sap IR Band Assignment

The spectral analysis of the ATR data of phloem sap showed three main spectral features: broad mixed band peaks were located in the range of 1700–1530 cm^−1^ (band 1); 1440–1325 cm^−1^ (band 2), and 1170–950 cm^−1^ (band 3) wavenumbers (WN), and differed in prominence and appearance according to their cluster association, as demonstrated in the spectral shading of the average sap spectrum (Figure 3c). In detail: band 1 consisted of three to four minor features at 1640, 1620, 1570, and 1515 cm^−1^, which were associated with clusters 6–8 of hca1. These band regions are well known as markers of nitrogen-containing substances such as proteins and amino acids (since bands of C=O and C-N stretching vibrations such as Amide I (1700–1600 cm^−1^) and N-H bending of Amide II (1580–1520 cm^−1^), both of which contribute to these band positions). The major band in clusters 3 and 4, around 1600 cm^−1^, was well situated within the N-H stretching region for primary amines (1640–1560 WN); band 2 was dominated by a strong, sharp peak at 1352 cm^−1^ and a broader peak at 1400 cm^−1^. Both could be associated with S=O stretching; the more pronounced could be associated with sulphates and the sharp band corresponded to the same feature in sulfonamides or sulfonates; band 3, again, was a mixed band with multiple dominant features including a dominant peak at 1080 and a shoulder around 1008 cm^−1^. P=O stretching vibrations generally occur around 1080 and C-O stretching in carbohydrates around 1000 cm^−1^; these are common associations for these bands.

Clusters 3 and 4 showed a more even distribution of the three main band regions, whereas clusters 6 and 7 were dominated by the sharp peak at 1352 of band 2 associated with sulfur-containing biomolecules. The absorbance value favors clusters 4 and 5 in respect to total absorbance and clusters 7 and 4 regarding the band at 1352 cm^−1^ as dominating features.

### 3.4. Sap MS Components

Phloem sap of *B. napus* is usually dominated by sucrose (main sugar) and Gln/Glu (main amino acids) [22]. It also includes other soluble components, the composition, specificity and relevance of which are under investigation [23]. To also cover the less abundant constituents (and their cluster formation within the vascular bundle), the applied chromatography/MS strategy was optimized accordingly. Untargeted metabolite analysis identified 77 compounds with the known annotation (as listed in Supplementary Table S4). Among them, many intermediates of sugar catabolism and glycolysis were detected, as well as organic acids, nucleotides, and cofactors. For 6 out of 77 metabolites, we detected variations in abundance higher than log2fold when comparing shaded versus control conditions (up: allantoate, 2-Ureido-Gly, phosphoenolpyruvate (PEP), UDP-glucose; down: trehalose-6-P, 2-oxoglutarate). Among all detected compounds, 37 were not coaligned with main infrared spectral components in their sample composition pattern. These features presented the most homogenic trend characteristic according to the sample set and were grouped in clusters 1 and 2 (Supplementary Table S3). Alignment is achieved when both a subset of IR components and MS compounds are clustered together. All other clusters generally aligned with one or more of the previously described IR bands and consisted of biomolecules containing the functional groups associated with these bands. Clusters 4 and 8 were strongly associated with the tricarboxylic acid (TCA) cycle, whereas clusters 6 and 7 had a strong link to glycolysis, energy metabolism, and resource management in general (e.g., amino acid metabolism and carbohydrate metabolism).

**Figure 4:**
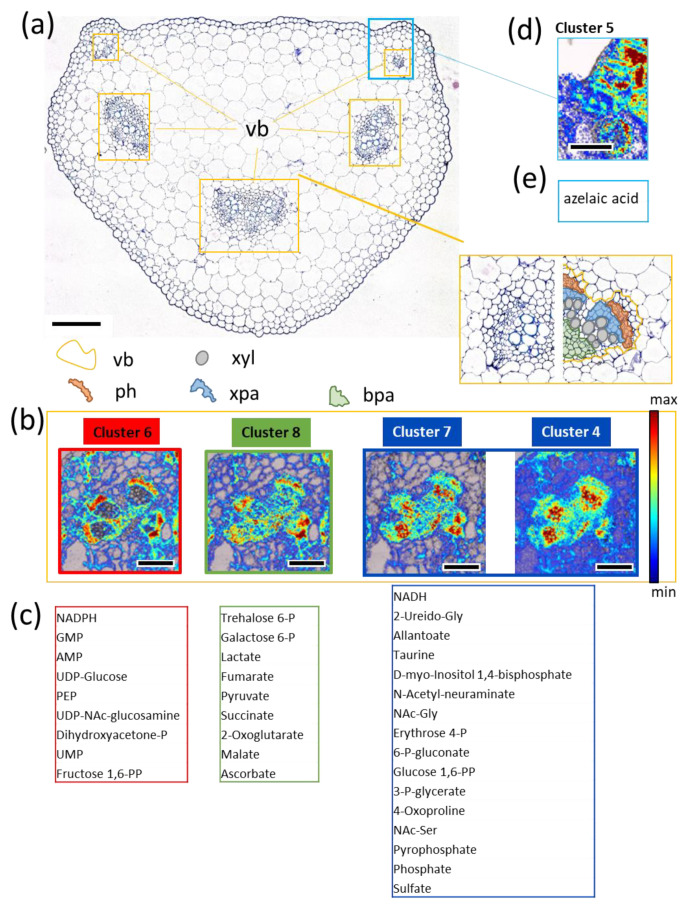
**Petiole architecture and cluster hot spot imaging.** (**a**) The represented petiole cross section shows the typical locations of the three major and the two minor vascular bundles (yellow boxes); the following tissue regions specific to the vasculature were defined (as marked by colored outlined areas): vb—vascular bundle; ph—phloem; xyl—xylem; xpa—xylem parenchyma, bpa—base parenchyma; (**b**,**c**) dedicated images for clusters 4, 6, 7, and 8 demonstrate the allocation in characteristic regions (**b**) of the vascular bundle and the associated MS components (**c**); (**d**,**e**) dedicated image for clusters 5 (**d**) and the associated MS components (**e**). Scale in (**a**) is 200 µm, and scales in (**b**,**d**) are 100 µm. IR images are absorbance color-coded from min (blue) to max (red) values. The transparency of the IR overlay is also linked to absorbance intensity with regions below the detection limit being transparent.

Representatives of groups (1 and 2) such as uric acid, threonate, and others may give an indication of their under representation. Because the method relied on dried extracts dissolved in methanol, the named compounds and their clusters were simply not evident in the spectral PCA loadings due to being poorly solvable to non-solvable in this environment. Although this was an essential step in order to avoid dominant water bands in the sap spectra, it likely caused spectral under representation of this group. Clusters 6 to 8 had the highest proportion of MS components associated with infrared fingerprints. As such, distinct spectral bands showed good representation of features associated with the underlying MS representatives. Most notably, this can be seen in the sulfonates and sulphate vibrations between WN 1400 and 1350 cm^−1^, which was a dominant feature of cluster 7′s fingerprint and an exclusive MS feature in this group. Other features were less distinctive in their spectral dominance. In most cases, associated compounds originated from similar metabolic pathways. Cluster 8, for example, showed a strong association with parts of the TCA cycle, whereas cluster 6 represented several members of the energy metabolism (Figure 4c). In conclusion, the assumption that correlated components are often associated with similar functions can be supported by this finding. From the perspective of chemical composition, this makes sense. Reactions, depending on specificity, rely on a similar pool of substrates to create similar chemical products. By this reasoning it makes sense to expect distinct fingerprint features of the infrared spectrum to align the same way with specific clusters of chemical compounds.

### 3.5. FTIR Imaging of Metabolic Clusters

Cellular composition (chemical trends) and metabolism (biochemical reactions) are often tissue specific. Different chemical environments favor different chemical reactions. As a result, functional regions that perform singular tasks based on a specific set of chemical reactions will result in specific accumulation/depletion patterns of underlying reaction products and substrates. When these patterns change over a certain time scale (or experimental setup), they will correspondingly be reflected in sample composition. Such patterns should be recognizable in samples such as phloem sap, as well as locally in imaging approaches, and can therefore be utilized in tracing back to the original regions. This approach is similar to the ways in which partial patterns of a fingerprint contain information to identify complete fingerprints from a database.

The six clusters that had IR features associated with them were introduced in the chemometric modeling routine to identify possible regions of origin in tissue samples of plant petioles. Figure 4a shows a characteristic cross-section of the respective tissue in *B. napus*. The most important distinct tissue features are the vascular bundles, as marked in the figure. In general, the petiole has three major vascular bundles, often consisting of three smaller subunits, and multiple off-branching vascular strains. Among the core tissue regions of a vascular bundle, marked as colored regions in Figure 4a, the most important are the phloem tissue region (ph, red) and xylem (xyl, grey), which, together, facilitate the transfer of assimilates, nutrients, and water between plant organs. Xylem is responsible for water transport while the much more compact phloem tissue transports organic metabolites such as sucrose within much smaller structures, called sieve tubes, and which are accompanied by companion cells responsible for loading and unloading of metabolites [24]. These tissues are embedded in a support tissue called parenchyma. For later comparisons the parenchyma was devided into xylem-associated (xpa, blue) and basal (bpa, green) tissue. In the upper left and right corner of the petiole cross section (Figure 4a), additional protrusions are located, which resemble the beginning of the leaf blade and tend to incorporate mesophyll tissue.

Within these tissues, all but clusters 1–3 displayed high and low abundance for the designated signals. Cluster 3 (responsible for only ~2% of the measured phloem sap absorbance) was generally not detectable in the measured tissue samples and was therefore omitted from further analysis. The EMSC model used a 10% subset of representative pixels as a reference set. These values give an estimation of the information content of an imaged feature. The reference set was generally modelled more accurately due to more limiting model constraints, such as non-negativity and upper absorbance limits. This provided us with an additional layer of quality control to evaluate the image data. The constrained linear least squares (LSQ) model can be compared with the regular partial least-squares (PLS) procedure used to compute all pixels. By comparing the values for both modeling approaches (LSQ~PLS) on the reference pixel set, the predictive capability of single compounds by Pearson correlation and signal-to-noise estimation could be determined. Cluster 3 was excluded because of poor R² values (0.45–0.75) in all investigated images and poor signal-to-noise ratios (0.11–0.22).

Representative images for the identified metabolic clusters are given in Figure 4b,d. The color scheme was based on the absorbance intensity of the cluster related IR fingerprint as established from the sap spectra principal components. They resembled the absorbance of the underlying pixel, which the EMSC model was able to explain with that component. Therefore, higher intensities showed likely regions of origin for the given cluster and its coaligned MS features. Looking back at the sap spectra, over 70% of the spectral information was explained by clusters 4 and 5. As shown in Figure 4b, clusters 6 and 8 were much more abundant in key vascular regions, explaining ~20% of the average image pixel, whereas cluster 5 seemed to be mostly disassociated with vascular structures. Remarkably, the only consistent increase above noise level for cluster 5 was associated with the ear-like tissue protrusions in the upper left and right corner of the petiole cross-section (Figure 4d). Sap clusters 4 and 6–8 were highly consistent with known functional regions of vascular tissue. Clusters 6 and 8 both peaked in the phloem regions, whereas cluster 8 was instead located elsewhere in the vascular structure, in particular the bpa region, and cluster 6 was almost completely void of the xpa region. Clusters 4 and 7 co-localized in the xylem parenchyma (xpa region, Figure 4). These four clusters represented approximately 32% of the average pixel absorbance.

### 3.6. Potential Biological Relevance of Chemometric Analysis and Metabolic Clusters in Vasculature

**Figure 5:**
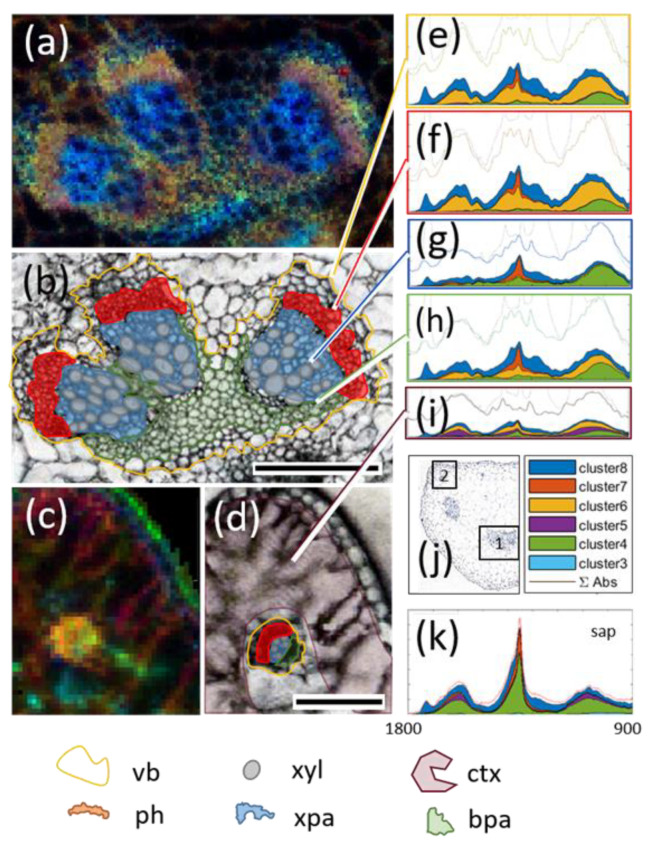
**Chemometrics of vascular tissues in petiole of *B. napus*.** (**a**) RGB image represents multicomponent analysis of the main vascular bundle (frame #1 in (**j**)). The red image channel is a representation of cluster 6, the green channel is associated with cluster 8, and clusters 4 and 7 are grouped in the blue color channel. (**b**) The corresponding visual images along with associated regions of interest; (**c**,**d**) the protruding tissue region associated with cluster 5 identity (frame #2 in (**j**)); (**e**–**i**) deconvoluted spectra of the marked regions with cluster contributions shaded according to the legend; (**k**) cluster related spectral features of sap. Abbreviations: vb—vascular bundle; ph—phloem; xyl—xylem; xpa—xylem parenchyma; bpa—base parenchyma; ctx—cortex/beginning mesophyll. Scale bars represent 100 µm.

Using the approach presented above, it became possible to display the associations of MS data (36 compounds) with functional regions of vascular bundles. In order to better illustrate the compartmentalization of the vascular tissue, the corresponding images were normalized to greyscale and combined as composite images using the red channel for cluster 6, the green channel for cluster 8, and the blue channel for cluster 4/7 (Table 1). The typical images, as in Figure 5a, showed a clear compartmentalization within the vasculature. Larger veins often grouped into three singular regions that were supported by base parenchyma (bpa, Figure 5a,b). In the image, this region is mainly green (cluster 8 dominated in the center) and gradually shifted to red in the direction of the phloem (ph region). The phloem area in particular was dominated by cluster 6, with a yellow shift towards the top, indicating a mix of clusters 6 and 8 in that region. Many of the co-located IR features (Supplementary Figure S3f) were associated with sugars (e.g., sucrose), amino acids, and bound water (a common observation in hydrophilic environments), which aligns well with the tissue function (assimilate transport). The border region, between the xylem parenchyma (blue colored, cluster 4 dominant) and phloem (red), was also of great interest. Blue was strongly co-localized with the IR signatures of proteins, as well as correlated with the NADH from sap (as derived from the MS data). Here, NADH, pyrophosphates (PPs) and phosphates (cluster 4/7, blue) and NADPH, GMP, AMP, UMP (cluster 6) was found to localize in close proximity but also separated in two groups. All of these compounds play important roles as co-factors in biosynthetic reactions of numerous metabolic pathways. This underlined the functional importance of these neighboring regions and emphasized the strict localization border between the two clusters. Vascular bundles of petioles comprise cambium tissue (thin layer of differentiating cells), which is similar to stem tissue. It is localized between the xylem and the outward-extending phloem tissue and the origin of both. The functional cell differentiation and the formation of cell wall structures are associated with carbohydrate metabolism and compounds such as cellulose, pectin, and hemicellulose [25]. Indicative of this process was the occurrence of cluster 6 (red) and the associated UDP–glucose at one side of the border between the xylem and phloem (blue/red, Figure 5). As proposed by Hoch [26], hemicelluloses and pectins not only have structural functions (as cell walls) but also provide a secondary carbon source when sugars and other backup sources (such as starch/fructans) are not sufficiently available. This is important for environmental adaptation and/or during shifts in the source/sink regime, and has proven effects on pectin and hemicellulose composition in *B. napus* [27]. Such processes in response to environmental or developmental changes would involve carbohydrate metabolism and essential components such as UDP-glucose, conveniently hinted at in this region.

For smaller veins (Figure 5c), the compartmentalization into phloem, xylem, and parenchyma tissue was not as clear due to the optical resolution limits of conventional IR microscopy. The section of petiole was close to the leaf base, so some tissues within the minor upper part of section comprised some chlorophyll-containing cells compared to the remaining part of section (Figure 5j, regions 1 and 2). Correspondingly, two distinct patterns emerged when comparing these different regions by their deconvoluted fingerprint spectra, as indicated in the subplot (Figure 5e–k). In the first region, the regional fingerprints (Figure 5e and Figure 4f) showed strong contributions of clusters 6 and 8 (as discussed above), whereas the fingerprint in the xylem parenchyma (xpa region) was defined by clusters 4 and 7. These clusters contribute to a mix of amino acid-derived components, as well as sulphates, and sugar-bound phosphates and PPs. The lower basal part of the parenchyma represented a mix of the aforementioned chemotypes.

The region closer towards the leaf base’s protruding ribs incorporated more and more green tissue (indicated as region 2 in Figure 5j), and fingerprint features became more homogenous (Figure 5i). A combination of the spectral fingerprints is displayed in Figure 5g and Figure 4i, and is most representative of the deconvoluted sap fingerprint (Figure 5k).

### 3.7. Multicluster Imaging Reflects the Functional Arrangement of Vascular Bundles

**Figure 6:**
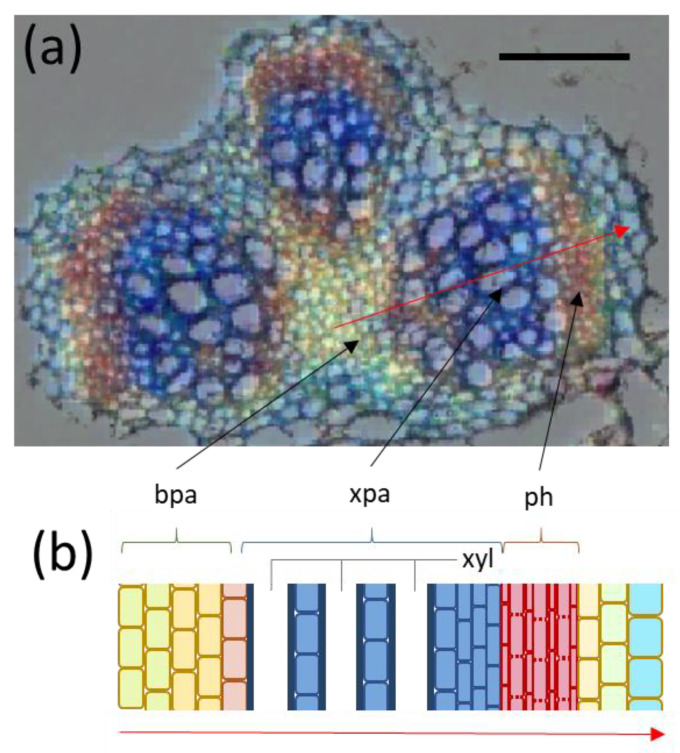
**Multicluster imaging of vascular bundles in *B. napus* leaf petioles**. (**a**) The multicomponent image (RGB) of the main vascular bundle overlaid on the tissue section. (**b**) The schematic cross section (along the red line in (**a**)) and color-coding; tissue identifier help to recognize how the color is associated with distinct tissues in the analyzed tissue section. Scale bar represent 100 µm. Abbreviations: ph—phloem; xpa—xylem parenchyma; xyl—xylem; bpa—base parenchyma.

This paper describes a novel conceptual approach on how to link information from infrared spectroscopic fingerprint, FTIR-imaging, and chromatography/mass spectrometry. The approach was exemplarily applied to the plant vasculature, which functions in the delivery of resources (sugars/amino acids/water/mineral nutrients) as well as in the provision of mechanical support [28]. Vascular bundles comprise different types of tissues such as parenchyma, xylem, phloem, proliferative tissues, and those providing mechanical support. Despite their complex structure and small size (few hundred µm), the approach enables defining metabolically distinct clusters and could demonstrate how they overlapped with structural and functional arrangements within the vascular bundle. The multicomponent image (Figure 6) displays that specific functional–structural regions are associated with specific metabolic clusters in the vascular bundle. The red image channel is a representation of cluster 6 (nucleotides, cofactors, and intermediates of glycolysis), which co-localizes with phloem cells. The green channel is associated with cluster 8 (organic acids and notably also the sugar signalling molecule trehalose-6P) and co-localizes with basal parenchyma (colored as yellow region in Figure 6 due to overlap with blue). Finally, clusters 4 and 7 (intermediates of amino acid and sugar metabolism) are grouped in the blue color channel, appearing in the xylem parenchyma. Such metabolic clustering results from corresponding metabolic activities in the respective tissues. Although a final explanation of these findings requires more detailed studies, this work clearly represents an additional step towards an improved understanding of the metabolic functioning of vascular bundles.

Rabe and coworkers [29] described another multimodal approach of FTIR-guided MALDI-MSI, where fingerprint analysis of IR images was utilized to identify regions of interest for the more complex MSI technique; thus they made use of a simple imaging technique to define the focal point for a complex, work-intensive, highly detailed technique. Within the approach, the correlation of sap components with IR features and their localization pattern creates an essential link in the overall interpretation of experimental findings that might otherwise be inaccessible. Although some component groups identified in the MS data were not associated with IR features, this was likely due to solubility limitations. Here, optimization potential can be sugested to allow for a better overall representation of component groups identified by MS. Moreover, additional chromatography modes could be integrated in future experiments to increase the number of metabolites. The more components are identified by MS, the higher the potential for linking spectral fingerprints to functional information. Overall, spectral fingerprint interpretation allows for access to many tissue/plant species applications, as well as other research backgrounds. It enables one to link localization with the high chemical identification potential of MS-based approaches.

## 4. Conclusions

The chemometric image correlation of IR and MS data (CHIC-IR/MS) help overcome some limitations of two technologies (MS and spectroscopy/imaging). A key advantage of assigning chemical clusters to possible target regions is that it allows a more comprehensive understanding of biological functionality. Given the presented approach is a first step, deriving scientific conclusions from this approach has to be carefully evaluated. There is still room for method improvement and refinement, both in experimental procedures and data analysis. Even so, the analytical concept may aid future synergistic research approaches leading to biotechnological innovations in plant research, as well as adapting to other research areas.

## Figures and Tables

**Table 1:** Overview of identified sap clusters and their image affiliations according to hierarchical clustering of tissue abundance. According to the hierarchical cluster analysis of distribution patterns demonstrated in Supplementary Figure S3, sap clusters 3–8 were associated with the depicted four image clusters and additional modelled infrared library features. The three most prominent image clusters were associated with the three color channels in RGB images as presented. Absorbance scores for IR features are given as relative to absolute pixel absorbance in the range of 1800–900 cm^−1^. The most prominent sap feature by absorbance and its associated MS components are presented in bold.

Image Cluster 1		Image Cluster 2		Image Cluster 3		Image Cluster 4	
**RGB Channel in Multi Component Images**							
blue		green				red	
sap cluster id	[abs/abs]	sap cluster id	[abs/ abs]	sap cluster id	[abs/abs]	sap cluster id	[abs/abs]
**cluster 4** cluster 7	**0.0768** 0.0185	**cluster 8**	**0.1068**	cluster 3 **cluster 5**	0.0019 **0.0115**	**cluster 6**	**0.1093**
correlated IR library features							
Total Protein Protein Type 1	0.0149 0.0082	2nd Metab.	0.0802	Cell wall	0.0291	Carb Soluble sugar Hexoses Bound water Amino acids 2nd Metab.	0.0873 0.0479 0.0285 0.0532 0.0756 0.0401
IC-MS sap features correlated with sap IR freatures							
**NADH** 2-Ureido-Gly Allantoate Taurine D-myo-Inositol 1,4-bisphosphate N-acetyl-neuraminate NAc-Gly Erythrose 4-P 6-P-gluconate Glucose 1,6-PP 3-P-glycerate 4-Oxoproline NAc-Ser Pyrophosphate Phosphate Sulphate		**Trehalose 6-P** **Galactose 6-P** **Lactate** **Fumerate** **Pyruvate** **Succinate** **2-Oxoglutarate** **Malate** **Ascorbate**		Allantoin Oxaloacetate Mevalonic acid-5P Isocitrate **Azelaic acid**		**NADPH** **AMP** **GMP** **UMP** **UDP-Glucose** **UDP-NAc-glucosamine** **PEP** **Dihydroxyacetone-P** **Fructose 1,6-PP**	

## Data Availability

The data presented in this study are available in supplementary Tables S1 to S4 and Figures S1 and S2. Library spectra for cell wall standards as dedicated to originate from INRA in Table S1 can be aquired from the histochem database after identification on the web application under https://pfl.grignon.inra.fr/shistochem/ (accessed on 20 August 2021).

## Supplementary Materials

The following are available online at https://www.mdpi.com/article/10.3390/biom11111717/s1: Figure S1. Sap library development. Figure S2. Derived spectral library after PCA. Figure S3: Selective analysis of color-coded regions in multi cluster images. Table S1: Overview of spectral library components and derived image variables. Table S2: Overview of normalized IR and MS scores. Table S3: Overview of MS feature association to sap clusters according to hca1. Table S4: Untargeted metabolite analysis.
