# Supplementary material for: Probing the Metabolic Landscape of Plant Vascular Bundles by Infrared Fingerprint Analysis, Imaging and Mass Spectrometry
Source: Biomolecules. 2021 Nov 18;11(11):1717. doi: 10.3390/biom11111717 (PMC8615794; doi:10.3390/biom11111717)
Supplement: Supplementary file 1 [file biomolecules-11-01717-s001.zip › Supplemental Fig 1_3.pdf]

Supplementary Figure S1. Sap library development. (a) A example of 6 normalized representative sap aliquote spectra. (**b**) The PCA loadings of the raw ATR sap spectra shows significant noise associated with water vapor bands (WN>1400), an experimental noise factor. (c) PCA derived library spectra (colored) and setup/baseline (exp) and non-fingerprint (nmir) related IR features in black. (d) Spectral deconvolution into noise and baseline features (black) and fingerprint related features (red) of the raw ATR spectra (colored) as determined by constrained linear least squares regression. (e) Baseline corrected ATR sap spectra after modelling with PCA 1 eigenvector library. (f) Second iteration PCA reveals some differences in the loading vectors as well as less noise in major components. (g) The library is extended by newly found eigenvectors and 2 distinct spectra explaining residual features (rf). (h) Remodelling of the baseline corrected spectra (colored) explains 99% of fingerprint (red) using the PCA derived library. Noise and nmir components are presented in black.

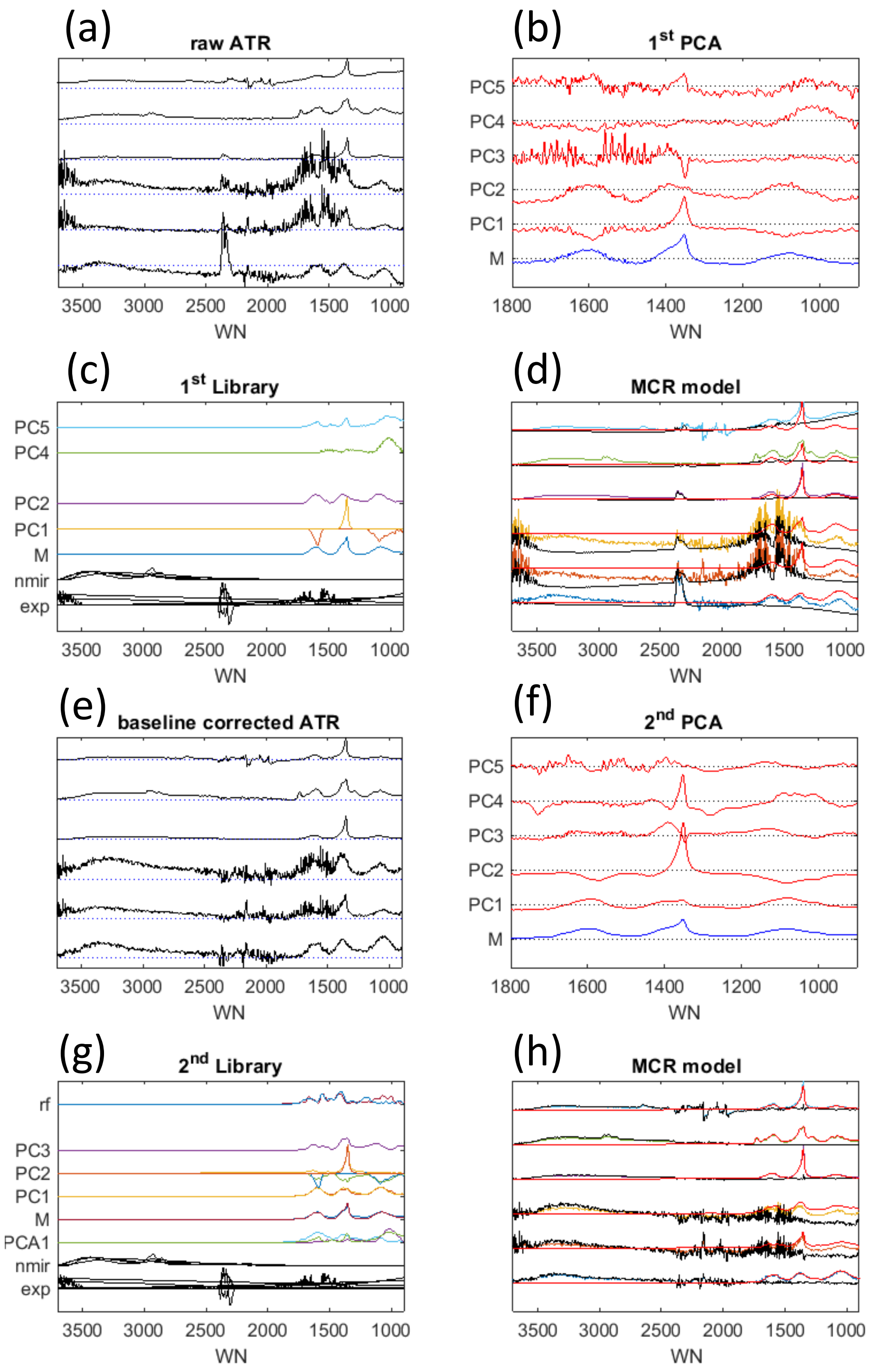

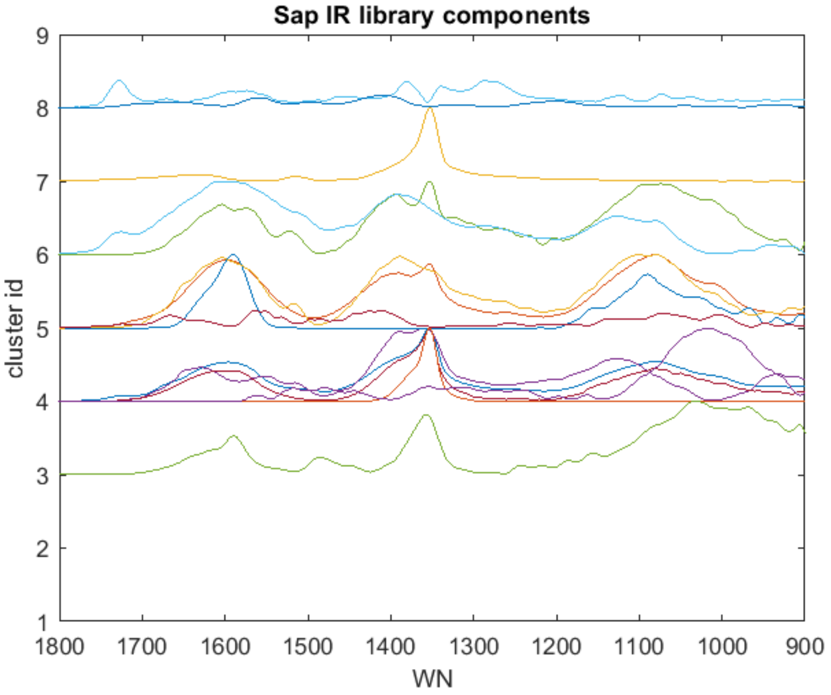

Supplementary Figure S2. Derived Spectral library after PCA.

The displayed spectra display the unique fingerprint features derived from denoising eigenvectors of the first iteration of PCA and reiterated eigenvectors after the second iteration. 15 Spectra are included in this library. According to hierarchical clustering they represent 6 out of 8 groups. Grouped spectra generally combine similar behaving eigenvectors of both iterations.

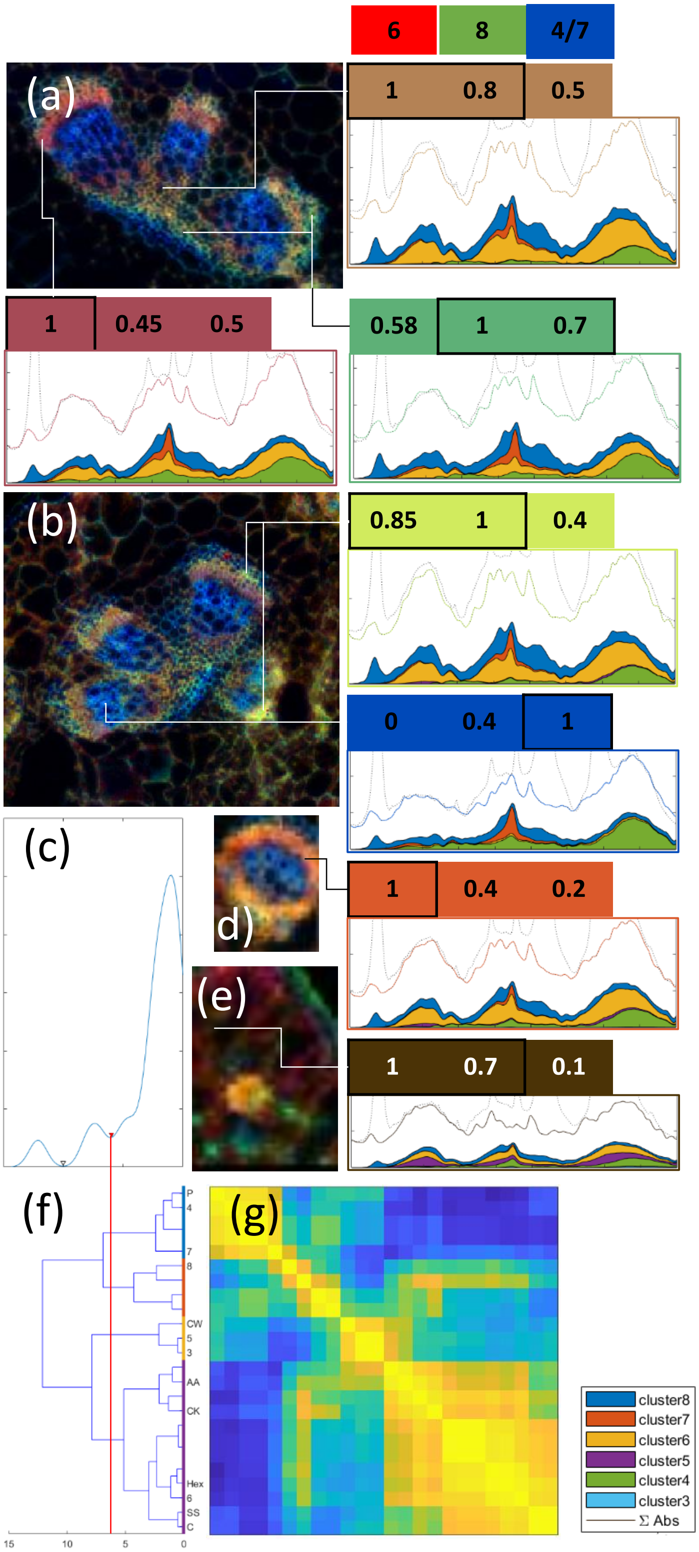

Supplementary Figure S3. Selective analysis of color-coded regions in multicluster images.

Explenation of the procedure: The multicomponent images (RGB) of various vascular bundles and adjacent tissues from the central vascular bundle outwards (**a,b,d,e**) allows the selective analysis of spectral cluster features according to the colour coding in the images.

The red image channel is a representation of cluster 6, the green channel is associated with cluster 8 and clusters 4 and 7 are grouped in the blue color channel. The color of selected areas is shown in the associated boxes with the weight of each color channel described by the numerical values (0-1) and the dominant color channel marked by the frame. The associated spectral features are displayed underneath each box and deconvoluted cluster features are marked according to the legend.

A hierarchical cluster analysis **(c, f**) of the selected regions shows cluster association along with additional spectral features of the EMSC model deconvolution. A cumulative density function of linking distances (d) reveals two minima relevant for clustering. The Pearson correlation matrix of the clustered IR components is given in (**g**).

Abbreviations: C - carbohydrate features; CW - cell wall carbohydrates; SS - soluble sugars; Hex - hexoses, AA - amino acids; P - proteins and numbers according to cluster id.
